# First report of a nearly complete comatulid crinoid (Comatulida, Echinodermata) from the Cretaceous of Australia

**DOI:** 10.1038/s41598-025-90111-2

**Published:** 2025-03-12

**Authors:** Mariusz A. Salamon, Tamas Kapitany, Bartosz J. Płachno

**Affiliations:** 1 Faculty of Natural Sciences, Institute of Earth Sciences, Laboratory of Palaeontology and Stratigraphy, University of Silesia in Katowice, Będzińska 60, 41-200 Sosnowiec, Poland; 2 National Dinosaur Museum, 6 Gold Creek Road, Nicholls ACT 2913, Canberra, Australia; 3 Faculty of Biology, Institute of Botany, Department of Plant Cytology and Embryology, Jagiellonian University in Kraków, Gronostajowa Street 9, 30-387 Kraków, Poland

**Keywords:** Marine biology, Palaeontology

## Abstract

Finds of stalkless comatulid crinoids in the Southern Hemisphere are extremely rare and are almost exclusively of their centrodorsals. Complete or nearly complete specimens (centrodorsal with articulated arms, cirri and/or pinnules) are known from only a few localities. Here we report a comatulid fossil comprised of a centrodorsal, arms, and pinnules, assigned to Solanocrinitidae gen. et sp. indet. The shape of the centrodorsal; the arrangement, shape, and size of radials; and the morphology of the radial cavity suggest similarity of this fossil to *Solanocrinites* or *Archaeometra* rather than to *Comatulina*, *Pachyantedon*, or *Palaeocomaster*. It cannot be ruled out that the studied specimen belongs to *Decameros* with its relatively low and discoidal centrodorsal. In the light of last phylogenetic analysis both *Solanocrinites* and *Decameros* were recovered as part of a clade comprising Himerometroidea, Tropiometridae, and Asterometridae + Ptilometridae. The preserved arms of the Australian specimen reveals variation in the branching pattern, that can be attributed to the inaccurate regeneration of arms after autotomy. This find represents the second articulated comatulid (except of uintacrinids) which consists not only of the centrodorsal, but also having more or less complete arms and pinnules, from the Cretaceous of the Southern Hemisphere. It is also the first found from Australia. The only opalized crinoid known in the world is the stalked isocrinid (Isocrinida) *Isocrinus australis*. Thus, the present find is also the first record of an opalized comatulid crinoid worldwide. Additionally, the thin sections reveal the occurrence of transversal sections of pelagic roveacrinids (saccocomids), which are also the first from the Australian continent.

**Supplementary Information:**

The online version contains supplementary material available at 10.1038/s41598-025-90111-2.

## Introduction

Comatulids (Comatulida) appeared in the Late Triassic and are highly diverse crinoids in recent marine ecosystems^1^. They shed their stalks during ontogeny and display high mobility (through crawling and swimming), a significant factor related to their success^2,3^. It should be noted, however, that among these crinoids there are also forms that retain stalks as adults. Examples include bourgueticrinids (Bourgueticrinina Sieverts-Doreck), thiolliericrinids (Thiolliericrinidae A.H. Clark), or guillericrinids (Guillecrinina Mironov and Sorokina) (e.g^1^. and literature cited therein). Among comatulids, the stemless crinoids with bizarre morphology - uintacrinids (Uintacrinida Broili), can be also distinguished. According to Rasmussen^4^ this crinoid group should be included in a separate order, but more recently Hess and Messing^1 ^included them in the order Comatulida. These crinoids do not resemble comatulids in the traditional sense (i.e., marked by the presence of a more or less pentagonal centrodorsal mostly covered by lateral cirri) - they have a globose calyx made of numerous plates without cirri (for more details see e.g^5^). This group, though reported from Australia (e.g^6^), will not be discussed in this article.

Comatulids are the only extant crinoid group that is globally distributed in both shallow- and deep-water settings^7^. Their centrodorsal serves as the interface between the cirri and arms, a major innovation in crinoid evolution. Any find of a complete or nearly complete comatulid is rare and deserves attention. Unfortunately, the large majority of fossil comatulid species were described based on isolated centrodorsals (e.g^1,8^). This in turn causes taxonomic problems as diagnostic features of extant forms also include morphologic features of ray branching pattern, cirri and pinnules. Salamon et al.^9 ^reviewed 39 Mesozoic–Neogene genera of comatulids and concluded that only seven taxa have been described based on both centrodorsal, cirri/cirarls and brachials/arms features. Moreover, Hess and Messing^1^ emphasized that in most of the intact comatulids, many morphologic features of centrodorsals are, unfortunately, partly hidden or indistinct. It is similar in the present case, where the basal part of the centrodorsal and the cirri are hidden in the rock matrix. On the other hand, the present find is one of the few Mesozoic comatulids known, where a fragment of the centrodorsal and arms and several pinnules are preserved.

## Remarks of geology and the age of Bulldog Shale

**Fig. 1:**
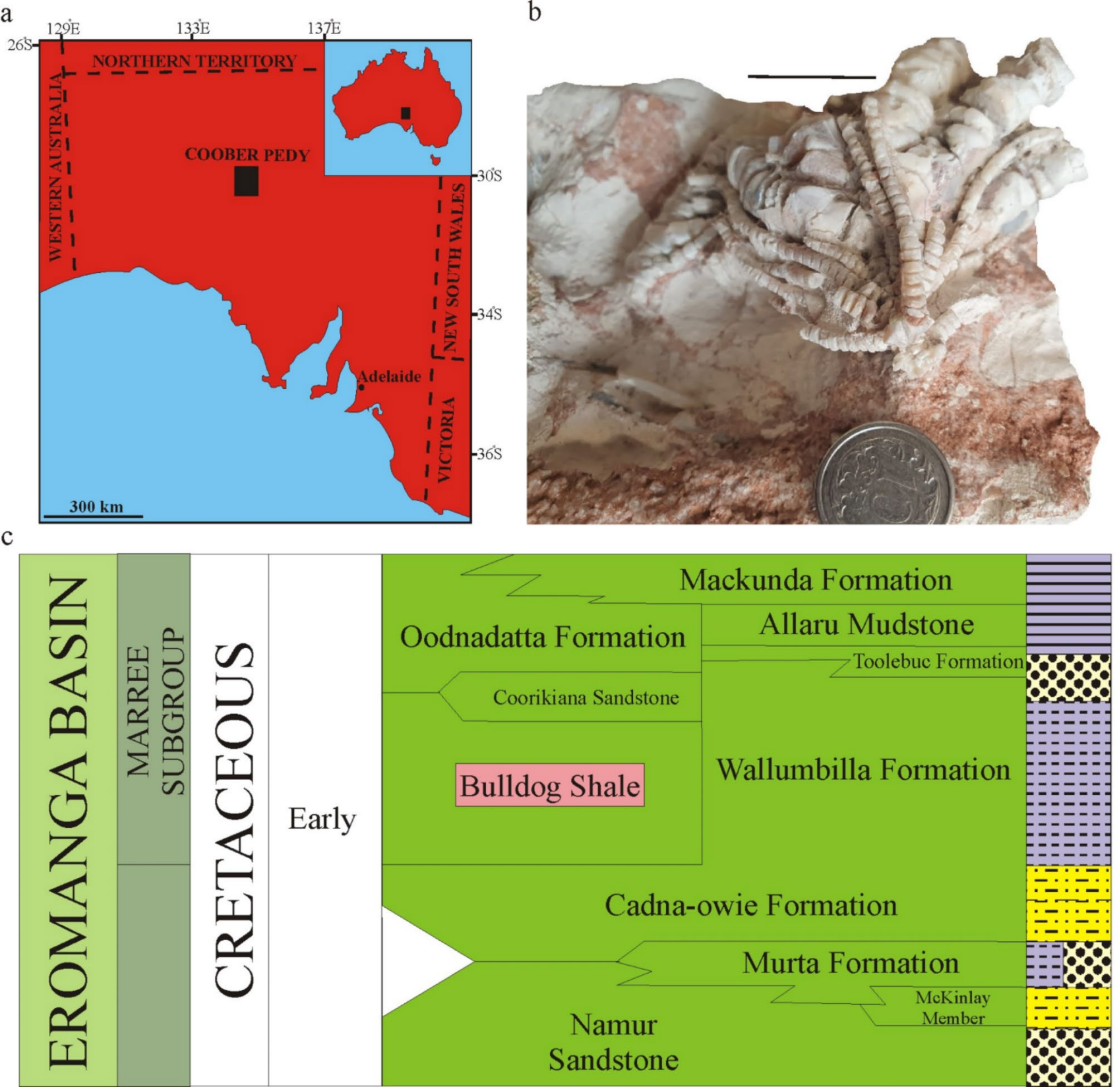
Location and stratigraphy of the investigated area and co-occurring with stemless comatulids stalked isocrinids. Map of Australia and Coober Pedy area (**a**) taken from^13^, slightly modified and simplified. Opalized *Isocrinus australis* Moore from Aptian of Coober Pedy area (**b**). Photo: BJP. Lithostratigrqaphy of Cooper/Eromanga basins in south Australia with position of the Bulldog Shale Formation (**c**) taken from Underground Water Impact Report Nappamerri Trough Natural Gas ATP 855^60^, slightly modified and simplified.

The most famous Australian sites with opalized fossils are the Coober Pedy and Andamooka (southern Australia); Fig. 1a. Herein, numerous fossils are found within the opal-bearing layers of the Cretaceous Bulldog Shale of Barremian to early Albian age^10^. Krieg and Rogers^11^ and Alexander et al.^12^, however, suggested an Aptian–early Albian age for the Bulldog Shale based on microfossils. According to Barrett et al.^13 ^an Aptian age is usually proposed for the opal-bearing strata on the basis of molluscan remains (see also^14,15^), and such an age is accepted herein. These sediments are composed of dark grey, silty and sandy, smectite-rich claystones with lenses of sands, limestones and occasional erratic boulders; Fig. 1c.

## Results

Exact location data of the find is unavailable as it was extracted by opal miners. We provide general information only: slab with crinoid comes from Old Zorba field, which is located ca. 6 km north-west of Coober Pedy airport.

The specimen characterized here is in the National Dinosaur Museum, Canberra, Australia, and have the acronym: NDM1235.

### Crinoid description

Numerous fragments of stems, arms, calyces and more or less complete individuals of isocrinids have been recorded from this site (e.g., Fig. 1b) and will be published elsewhere.

Details of the comatulid description is provided below. Systematic description and terminology followed herein is after Hess and Messing^1^.

Order Comatulida A.H. Clark^16^.

Suborder Comatulidina A.H. Clark^16^.

Superfamily Solanocrinitoidea Jaekel^17^.

Family Solanocrinitidae Jaekel^17^.

Solanocrinitidae gen. et sp. indet.

#### Material

Slab with nearly complete specimens (with five arms preserved, excluding distal arm parts, with some pinnulars preserved and without visible cirri) stored in the collection of the National Dinosaur Museum, Canberra, Australia, and have the acronym: NDM1235.

#### Measurements

Centrodorsal diameter in proximal part: 11.53 mm; centrodorsal height: ca. 6 mm (comp. Supplementary material: Movie 1, 2); arm thickness: 3.75–7.80 mm; max. arm lenght: 73.10 mm; pinnules diameter: 0.13–0.34 mm; pinnulars lenght: 24.20 mm.

#### Description

**Fig. 2:**
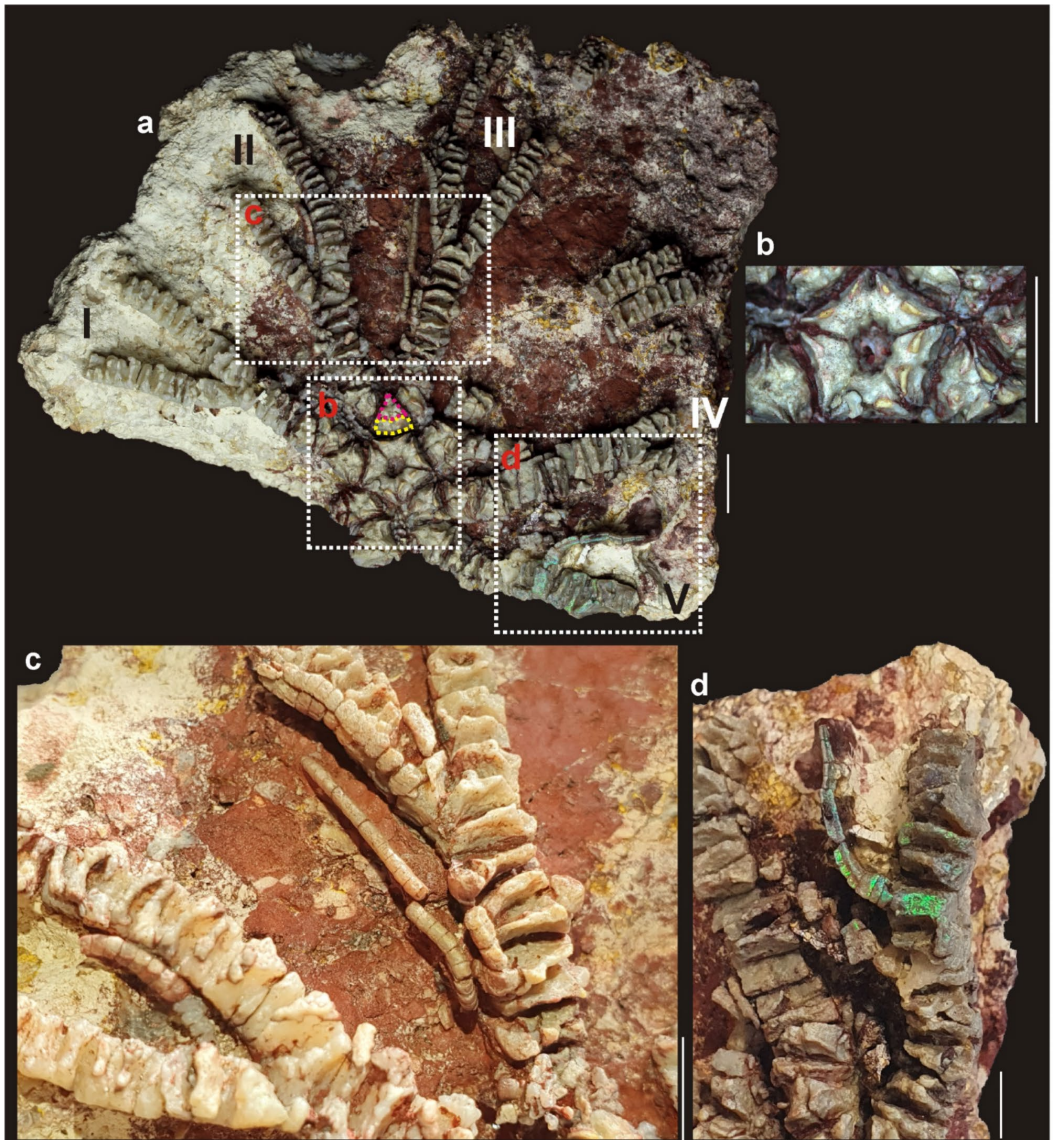
Solanocrinitidae gen. et sp. indet. from the Aptian of Bulldog Shale Formation, southern Australia. Scale bar equals 10 mm (a, c, d) and 10 μm (b). (**a**) Specimen with centrodorsal, arms and pinnules. Yellow dotted line – first primibrachial, pink dotted line – second primibrachial. (**b**) Enlargement of the centrodorsal. (**c**,** d**) Magnification of secundi-, tertibrachials and pinnulars.

**Fig. 3:**
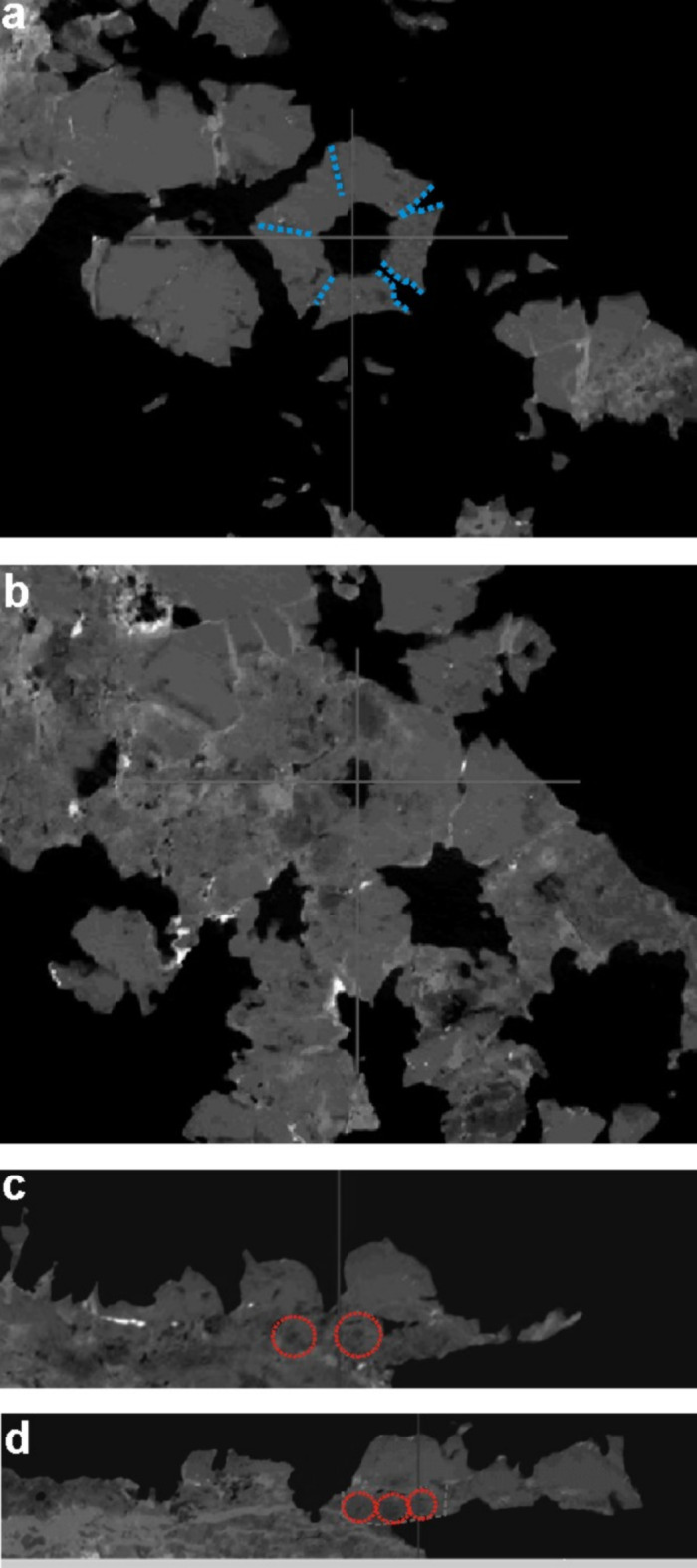
Solanocrinitidae gen. et sp. indet. from the Aptian of Bulldog Shale Formation, southern Australia. Cropped from supplementary movie 1 and 2. Not to scale. (**a**) Photo cropped from a scan taken from the proximal part of centrodorsal. Blue dotted lines - boundaries between radials. (**b**) Photo cropped from a scan taken from the proximal part of centrodorsal. Middle part of a centrodorsal. (**c**) Photo crapped from a scan taken from the side. Middle part of a centrodorsal. Red circles - cirrus sockets. (**d**) Photo crapped from a scan taken from the side. Lower part of a centrodorsal. Red circles - cirrus sockets.

Centrodorsal is five-sided (Figs. 2a and b and 3a and b). It is also more or less discoidal which is only visible on X-ray computed tomography images (Fig. 3c, d). Similarly with cirrus sockets that are circular and moderately large (Fig. 3c, d). Radials are massive and well-exposed. They are distinctly overhanging (Fig. 2a, b). Interarticular ligament areas are low. Adoral muscle fossae are also low and form triangular areas separated by a median notch. Radials gently slope towards a round radial cavity (Fig. 2a, b). The radial cavity is moderately wide, but very deep (Fig. 2a, b). Five massive arms are preserved, although the shape of the second primibrachial (= IBr2) (see pink dotted line on Fig. 2a) and the preserved proximal arm parts suggest that the individual had 10 arms. Arms are biserial (Fig. 2a–c). Rays are divided at primibrachial 2 (see pink dotted line on Fig. 2a). First primibrachial (= IBr1) is slightly lower than IBr2. The lBr2 is axillary and nearly triangular. First secundibrachial (IIBr1) is distinctly taller than the second (IIBr2). Every preserved arm branches at a different point. Arm marked as “I” (see Fig. 2a) branches at IIBr8; arm “II” at IIBr10; arm “III” at IIBr12; arm “IV” at IIBr14; and arm “V” consists of 13 secundibrachials and is still unbranched (for sumamry see Table 1). It is impossible to determine the specific type of articulation (muscular or syzygial). First pinnules are located on IIBr5. Pinnulars are circular to oval in cross-section, smooth and without comb-like structures (Fig. 2a, c, d). The most complete pinnule consists of 25 segments. Both proximal, medial, and distal pinnulars are of the same height; in case of distal pinnulars, their diameter decreases slightly (Fig. 2c, d).

**Table 1:** Number of brachials per arm segment.

	Arm no. 1	Arm no. 2	Arm no. 3	Arm no. 4	Arm no. 5
Secundibrachials	8	10	12	14	13
Tertibrachials	13 and 14	12 and 18	17 and 12	3 and 3	N/A
Quartibrachials	N/A	N/A	5 and 10	N/A	N/A

#### Remark

The number of secundibrachials is oddly variable within a single individual, which indicates that the branching patterns should not be treated as a diagnostic taxonomic feature. Oji^18^ observed the same phenomenon in the number of primibrachials of the stalked crinoid *Metacrinus rotundus *and concluded that most of the variability can be attributed to inaccurate regeneration of arms after autotomy caused by predation. On the other hand, it cannot be ruled out that this process does not have to be related to predation. Shibata and Oji^19^ pointed out that during the ontogeny of comatulids, there is programmed autotomy not related to predation, as a result of which the total number of arms increases.

*Discussion. *Based solely on the morphology of the distal (oral) part of centrodorsal, the Australian specimen is classified as Solanocrinitidae gen. et. sp. indet. According to Hess and Messing^1^ family Solanocrinitidae includes five genera (*Solanocrinites* Goldfuss, *Archaeometra* Gislén, *Comatulina* d’Orbigny, *Pachyantedon* Jaekel, and *Palaeocomaster* Gislén) recorded from the Lower Jurassic (Hettangian) to the Upper Cretaceous (Coniacian?) of Northern Hemisphere (Europe). The shape of the centrodorsal, arrangement, shape, and size of the radials, and the form of the radial cavity suggests that it is most closely related to *Solanocrinites* or *Archaeometra*. According to Goldfuss^20^, Gislén^21^, Rasmussen^6^, and Hess and Messing^1^, the centrodorsals of these genera are moderately high discoidal, truncated conical, and like the Australian specimen more or less 5-sided. Hess and Messing^1^ additionally expressed the opinion that the centrodorsal of *Archaeometra* is similar to *Solanocrinites*, but the adoral diameter is approximately twice that of the aboral apex. They are also distinguished by the way of arrangement, size, and number of cirrus sockets. At present this cannot be determined. The Australian material certainly cannot be associated with *Comatulina *d’Orbigny, whose centrodorsal is truncated conical to subhemispherical. Its adoral side has irregular furrows around a cavity. Additionally, the radials of this taxon are not so massive and well exposed as noted in Solanocrinitidae gen. et. sp. indet. (compare with Fig. 40/2 in Hess and Messing^1^).

The centrodorsal of another solanocrinitid, *Pachyantedon*Jaekel, is not known. This crinoid, found as an impression in a flint boulder, presumably in the Upper Cretaceous of northern Germany, was interpreted by Jaekel^22 ^as a comatulid with ten arms. Rasmussen^4 ^rejected this name, but Hess and Messing^1^ argued that this form resembles specimens of *Solanocrinites*, and therefore decided to include it in the family Solanocrinitidae. Although the centrodorsal of *Pachyantedon* is not known, it certainly cannot be related to the Australian specimen, which has branching arms (which *Pachyantedon* lacks). Additionally, in the latter the brachials, in contrast to Australian specimen, are strongly wedge-shaped.

The last representative of solanocrinitids, *Palaeocomaster* Gislén, is clearly different from the currently described individual. The shape of its centrodorsal may be 5-sided, but the exposed surface of radials is very low or more often concealed within a bulging centrodorsal that is most often rugose. Additionally, the radials slope steeply into the central cavity, which in this genus is very extensive. The last occurrences of this genus are known from the Tithonian of Austria, France and Switzerland.

On the other hand, in a recent study by Saulsbury and Zamora^23^, they performed a phylogenetic analysis in the light of which *Solanocrinites depressus* (d’Orbigny), *Decameros ricordeanus* Rasmussen, and *D. wertheimi* Peck and Watkins were recovered as a monophyletic clade. This tree topology accords with Rasmussen’s^6^ classification of *Decameros* as a subgenus of *Solanocrinites* and goes against more recent definitions of the Decameridae Rasmussen and Solanocrinitidae. *Decameros* and *Solanocrinites* are recovered as part of a clade comprising Himerometroidea A.H. Clark, Tropiometridae A.H. Clark, and Asterometridae Gislén + Ptilometridae A.H. Clark. Additionally, the stratigraphic range of *Decameros*would be consistent with the age of the present find (Valanginian–Albian; Hess and Messing^1^). Although the morphology of the basal part of the centrodorsal and cirrus sockets of the Australian specimen is poorly known only from X-ray computed tomography (Fig. 3), it follows that the centrodorsal in lateral view is rather low and discoidal, which is typical for *Decameros* (comp. e.g., Fig. 1A–C in Saulsbury and Zamora^23^).

*Distribution.* Lower Jurassic–Upper Cretaceous of Europe (Algeria, Czechia, England, France, Germany, Poland, Portugal, Spain, Switzerland).

## Discussion

### Mesozoic stemless comatulids

**Fig. 4:**
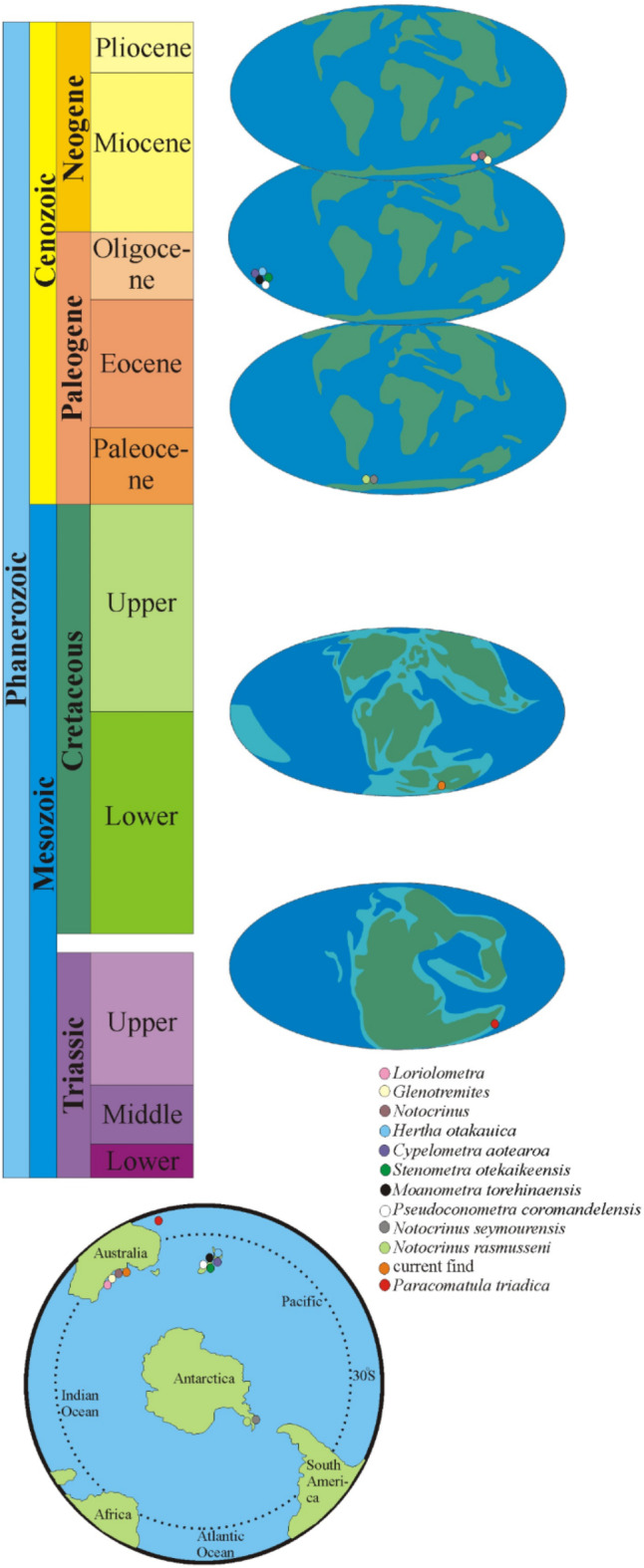
Post-Paleozoic distribution of comatulids within the Southern Hemisphere. Data taken from^1,9,24–30^.

The oldest and the only comatulid recorded from the Southern Hemisphere (and the oldest recorded in the world) is *Paracomatula triadica* Hagdorn and Campbell from the Late Triassic (Norian) Bouraké Formation of New Caledonia (Fig. 4). Hagdorn and Campbell^24 ^noted that this primitive form with short, 10-armed, branching once isotomously, and having long cirrals and tall brachials, is an offshoot of holocrinids rather than the more specialized pentacrinids (for details see^9,24^).

### Post-mesozoic comatulids

The first records of stalkless post-Mesozoic comatulids of the Southern Hemisphere are known from the Eocene (Ypresian–Bartonian; see Fig. 3in Whittle et al., 2018). Meyer and Oji^25 ^characterized seven specimens, none of which complete, from the La Meseta Fm. from Antarctic Peninsula. In two specimens, the centrodorsal and cirri were poorly preserved; in the other five, parts of arms and pinnules were preserved but the centrodorsal was broken away. Subsequently, from the same formation, Baumiller and Gaździcki^26^ described two centrodorsals, one with articulated arm segments and the other with several cirri and the most proximal brachials. They included the latter in a new species *Notocrinus seymourensis* (Fig. 4). This form, characterized by conical to subconical centrodorsals with the dorsal end not visible, was distinguished by its diminutive size (with a maximum width of 2.6 mm and height of 2.4 mm). Baumiller and Gaździcki^26^ reached the conclusion that *N. rasmusseni*, described by Meyer and Oji^25^ from the same formation, differs significantly from *N. seymourensis*. Size was regarded as the most significant feature, with *N. rasmusseni* being considerably larger than *N. seymourensis*. In case of the former, the distance between IBr1-2 and IIBr3-4 was 5.0 mm, whereas in the latter it was 2.5 mm. Additionally, variations in the morphology of the primibrachials were observed (for details see p. 111 in^26^).

A new family Pseudoconometridae (comprising a single genus *Pseudoconometra* with *P. coromandelensis*as type species) erected by Eagle^27^ can be distinguished by the presence of five radial pits on the adoral surface of the centrodorsal. The conical centrodorsal of a crinoid belonging to *P. coromandelensis* was documented in the late Oligocene (Chattian) of Waitete Bay in New Zealand (Fig. 4). Within the same sediments, Eagle^27^ documented another genus (*Moanametra* Eagle with *M. torehinaensis*as type species) included by Hess and Messing^1^ in the family Conometridae Gislén and noting that this taxon is based on single specimen and appears to be very similar to *Amphorometra*Gislén, belonging to Conometridae. The same author^28^ distinguished three new species of comatulids from the Oligocene (Chattian) of Otekaike Limestone Formation, New Zealand (Fig. 4). These were: *Stenometra otekaikeensis* Eagle, *Cypelometra aotearoa* Eagle, and *Hertha otakauica* Eagle.

Eagle^29^, identified a new family, Maorimetridae Eagle, with two new genera *Maorimetra* Eagle and *Zelandimetra* Eagle, and six new species of comatulids. These included the genera: *Amphorometra*, *Comaster* Aggasiz, *Maorimetra*, *Palaeantedon* Gislén, *Vicetiametra* Malaroda, and *Zelandimetra*, also from the late Oligocene (Chattian) strata of Otekaike Limestone Formation, New Zealand. However, it appears that both the new family and the genera proposed by Eagle^29 ^belong to the existing family and genera, as Hess and Messing^1^ did not include them in the revised *Treatise on Invertebrate Paleontology*.

Recently, Whittle et al.^30^ documented centrodorsals of three representatives of comatulids (*Notocrinus* sp., *Loriolometra* sp., *Glenotremites* sp.) from the South Australian Mannum Formation (Miocene, Aquitanian; Fig. 4).

### Records of free-swimming roveacrinids (Roveacrinida) from the Southern Hemisphere

Kristan-Tollmann^31^ noted occurrences of genus *Osteocrinus*Kristan-Tollmann (family Somphocrinidae Peck) in the Middle Triassic (Ladinian)–Upper Triassic (Carnian) of Europe (Austria, Italy, and Romania), and Asia (Afghanistan, China, Nepal, Timor, and Turkey). Timor, presently, is in the Southern Hemisphere, but in the Aptian (Cretaceous), it formed part of the Northern Hemisphere (see map 27 in^32^).

**Fig. 5:**
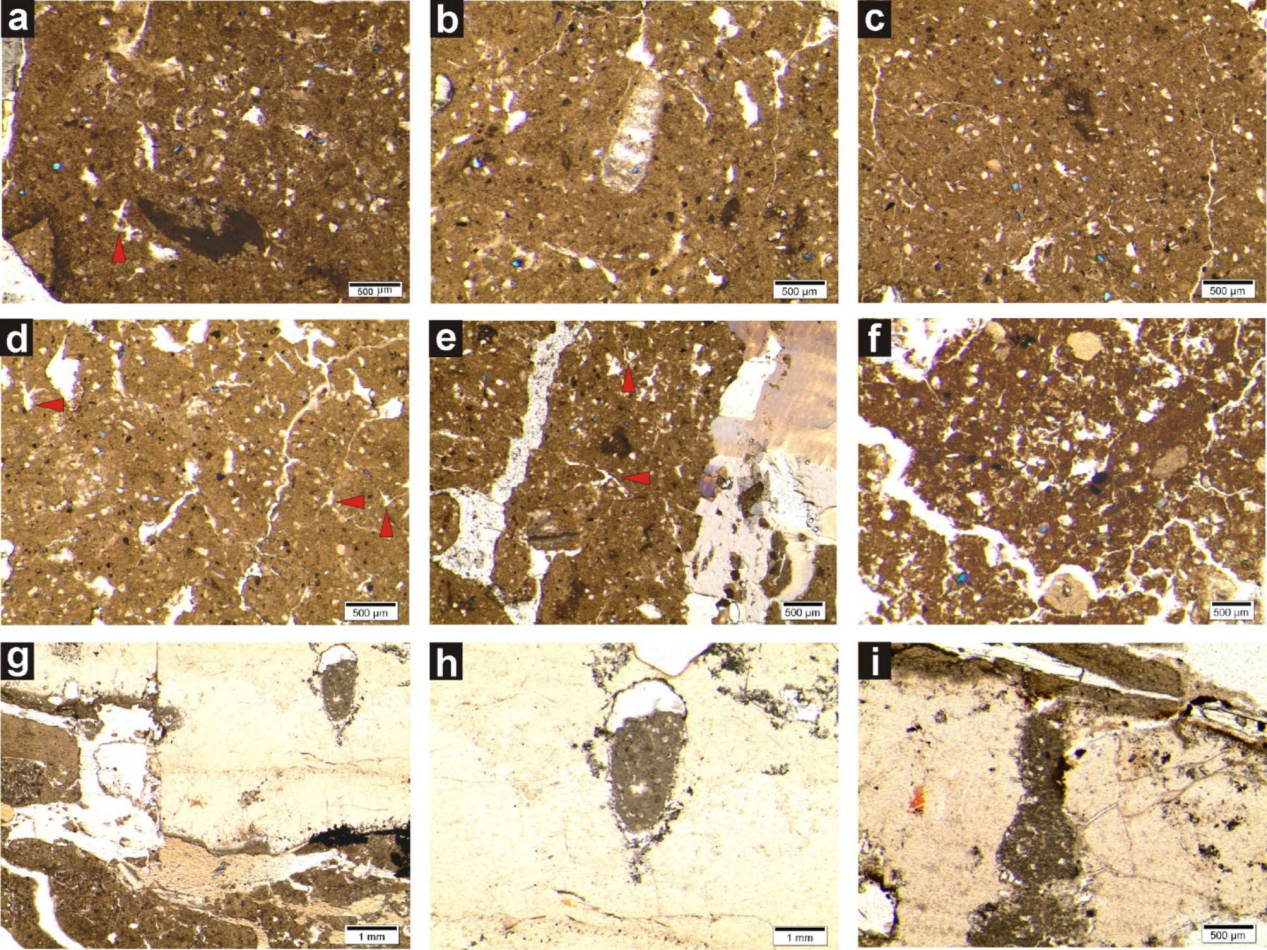
Microfacies from the Bulldog Shale of Coober Pedy in southern Australia. Scale bar equals 500 μm for (**a**–**f**,**i**), and 1 mm for g, h. Red arrows show free-swimming crinoids (roveacrinids, saccocomids; Roveacrinida Sieverts-Doreck, Saccocomidae d’Orbigny) which have never been previously reported from the Australian continent. (**a–d**) Silicified fine bioclastic wackestone. Silicified bioclastic wackestone with foraminifera and fine unrecognizable bioclasts. In micritic matrix fine quartz grains (**a**). Silicified fine bioclastic wackestone. In mitritic matrix numerous fine bioclasts and quartz grains (**b**). Silicified bioclastic wackestone. In micritic matrix quartz grains and “organic remains” (**c**). Sillicified bioclastic wackestone with algae remains, foraminifers and “organic remains” (**d**). (**e–h**) Silicified bioclastic floatstone/wackestone. Silicified bioclastic floatstone/wackestone with fragments of crinoids (on the right). In micritic matrix fine bioclasts, “organic remains” and fine quartz grains (**e**). Silicified peloidal-bioclastic wackestone/packstone with numerous micritic peloids, bioclasts and foraminifers (**f**). Silicified bioclastic floatstone with bored remains of crinoides. Borings filled with fine bioclastic wackestone (**g**). Bored crinoids filled with bioclastic wackestone (**h**). (**i**) silicified peloid-bioclastic wackestone/packstone showing bored crinoids.

The roveacrinids have been noted from only two Southern Hemisphere countries so far (Angola and Brazil), and herein, from the Aptian of Bulldog Shale, Australia (as identified from thin sections; see Fig. 5a,d,e).

*Roveacrinus* sp., *R. communis* Douglas, *R*. cf. *communis*, *R*. aff. *geinitzi* Schneider, and *R. pyramidalis *Peck have been reported from the late Aptian of Angola^33^. There are several studies from Brazil, but, like the present one, all are based on thin sections (see^34^ and references therein). Recently, Poatskievick Pierezan et al.^34^ from the upper Aptian and lower Albian of the Sergipe-Alagoas Basin (NE Brazil), recorded numerous cups and brachials and of a new roveacrinid genus and species (*Sergipecrinus reticulatus*). Genus *Sergipicrinus* was assigned to the subfamily Plotocrininae. Later, Poatskievick Pierezan et al.^35^ from the Maastrichtian of the Gramame Formation (Pernambuco-Paraíba Basin, NE Brazil) recorded abundant specimens of Saccocomidae and Roveacrinidae families within the Applinocrininae and Hessicrinae subfamilies (respectively).

### Palaeoenvironmental interpretation

The origins of the formation of the Australian opal fields, which provide as much as 95% of the world’s opal production, is still debated (e.g.,^10,36^). There is controversy over the age of the Australian opal, which is herein considered to be Aptian based on mollusks fossils (see above), and the source of silica, the composition of the opalizing fluids, and the formation mechanism of the precious opal. Jones and Segnit^37 ^and Kalinin and Serdobintseva^38 ^proposed the deep weathering model for the formation of opal. According to this model, opal formed slowly by the downward percolation and interaction of meteoric water with rock in an arid to semi-arid environment. The second syntectonic model was proposed by Pecover^39^. It involves the rapid formation of opal during hydraulic extension-related fracturing by silica-rich fluids under pressure. While this issue cannot be resolved herein, it is known that the mid-Cretaceous Australian opal deposits mined in the central-eastern and north-eastern parts of the continent were formed in a variety of environmental conditions, ranging from fluvial through coastal paralic to shallow marine and deep? marine^40^. So far, most attention has been paid to the opalized vertebrates occurring within these sediments, which are represented by the remains of numerous and diverse fishes. These and other opalized fossils include: aspidorhynchid scales, lamniform chondrichthyans teeth, and tooth plates of ceratodontiformes, shell fragments, limb elements and vertebrae of turtles, crocodylomorphs, plesiosaurs, pterosaurs, and dinosaurs. Among these, the most common are the isolated ornithopod remains. The rare opalized vertebrate bones come from frogs and mammals. Among the invertebrates and protists, there are marine and freshwater forms. The most common are foraminifers, belemnitids, gastropods, bivalves, and astacoid crustacean gastroliths. Among the articulated echinoderms documented so far are asteroids, ophiuroids of the genus *Ophioglyphoida*, and stalked crinoids *Isocrinus australis*. Moreover, every year tons of opalized plant material are excavated, especially wood fragments and pine cones. It is shown by the opalized fossil record that during the mid-Cretaceous, the forests were comprised of fungi and lichens, mosses, horsetails, ferns, but mostly by gingko trees and conifers. Fragments of opalized wood are common. The opalized plant material, outside the marine environment, indicates a grass-free ecosystem in which the opals were formed (e.g.,^13,41–58^ and literature cited therein).

Thin-section analysis of sediments from the Bulldog Shale of Coober Peddy are from three microfacies types: (i) silicified fine bioclastic wackestone (Fig. 5a–d), (ii) silicified bioclastic floatstone/wackestone (Fig. 5e–h), and (iii) silicified peloid-bioclastic wackestone/packstone (Fig. 5i). The silicified micritic matrix is ​​dominated mostly by unrecognizable fine and large bioclasts. In studied samples, foraminifers, algae remains, crinoids and unrecognizable remains were observed among the numerous bioclasts. Among the peloids, biotic origin rounded elongated or oval dark-colored micritic fecal pellets and irregularly shaped, rounded micritic algal-origin peloids are observed (cf^59^). Furthermore, dispersed fine quartz grains and possibly “organic remains” were commonly observed within the micritic matrix. Borings filled with fine bioclastic wackestone were commonly observed on crinoids skeletons. Microfacies analysis currently conducted on samples from Coober Pedy suggests a shallow marine water, mud-bioclastic dominated inner platform setting (restricted/semi-restricted lagoon) with a limited but continuous supply of material from land.

## Materials and methods

The slab with this comatulid crinoid was found in 2014 by Tim and Sam Seekamp and Pep Kovacik and was delivered by TK from Australia to the Palaeontological Laboratory of the Faculty of Natural Sciences of the University of Silesia in Katowice, Poland, in September 2024, for a detailed investigation. First, the individual was scanned with the X-ray computed tomography (CT-scanning) using a GE Phoenix v|tome|s at the Institute of Biomedical Engineering, Faculty of Science and Technology, University of Silesia in Katowice, Poland. It was investigated using different settings (voltage: 140 kV, amperage: 90 A, exposure time: 333 ms, projections: 2000, voxel resolution: 5 μm). Raw two-dimensional X-ray data were then processed using Phoenix datos|x. The movie was prepared using the following application VGStudio Max 2.0 (see Supplementary material: Movie 1, 2). The next step was to take macrophotographs of the specimen using a Canon Eos 7D digital camera equipped with macro lens EFS 60 mm and Samsung Galaxy S10 5G (Fig. 2a) and a Canon Eos 350D digital camera (Fig. 2b–d).

Thin sections were prepared at the Faculty of Natural Sciences, University of Silesia in Katowice, Sosnowiec, Poland. They were used for facies/microfacies analyses and photographed using a Nikon Eclipse E400 microscope equipped with a digital Nikon Digital Sight DS-Fi2 camera housed at the Department of Plant Cytology and Embryology, Institute of Botany, Faculty of Biology, Jagiellonian University in Kraków, Poland.

## Electronic supplementary material

Below is the link to the electronic supplementary material.

Supplementary Material 1

Supplementary Material 2

## Data Availability

All data generated or analysed during this study are included in this published article and its supplementary information files.
